# *JAG1* and *THBS2* Mutations in a Child Presenting With Incomplete Alagille Syndrome

**DOI:** 10.1097/PG9.0000000000000338

**Published:** 2023-07-17

**Authors:** Marion Almes, Antoine Gardin, Anne Davit-Spraul, Jérôme Bouligand, Dalila Habes, Emmanuel Jacquemin

**Affiliations:** * Pediatric Hepatology and Liver Transplantation Unit, National Reference Centre for Biliary Atresia and Genetic Cholestasis, FILFOIE, ERN RARE LIVER; † Inserm U1193, Hepatinov, University Paris-Saclay, Orsay, France; ‡ Biochemistry Unit; § Molecular Genetics and Pharmacogenetics, Bicêtre Hospital, Assistance Publique – Hôpitaux de Paris, University Paris-Saclay, Le Kremlin-Bicêtre, France

**Keywords:** cholestasis, next generation sequencing, thrombospondin 2, modifier gene

## To the Editor:

Alagille syndrome (ALGS) is an autosomal-dominant multisystem disorder mainly caused by mutations in *JAG1* (1,2). Phenotypic expressivity is variable and *JAG1* mutations have been found in patients with incomplete ALGS presenting with <3 major features of the syndrome (3,4). Thus, it is also useful to look for a genetic cause in such ALGS-like patients. A genome-wide association study performed in ALGS patients with *JAG1* mutations identified a significant locus upstream of the thrombospondin 2 gene (*THBS2*) (2). THBS2 regulates cell fate and angiogenesis and is expressed in bile ducts in mouse and human livers (2,5). THBS2 modifies JAG1–NOTCH2 interactions in vitro and a THBS2 SNP is associated with cardiovascular diseases (2,6,7). Therefore, THBS2 could be a candidate genetic modifier in ALGS patients, by disrupting JAG1–NOTCH2 signaling (2). So far, *THBS2* mutations have not been reported in ALGS patients.

**FIGURE 1.**
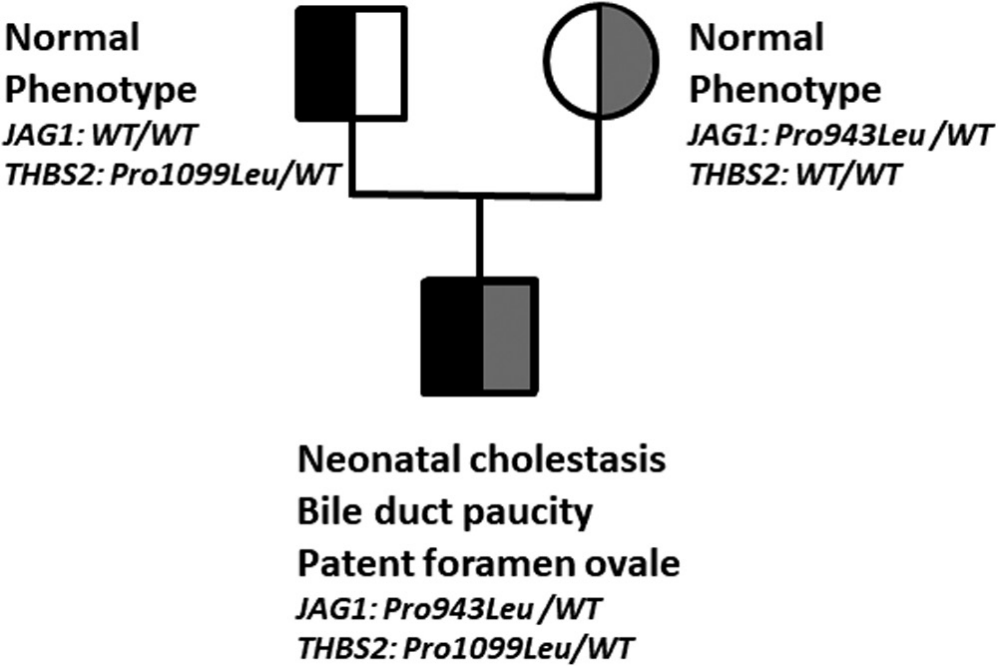
Pedigree and mutation analysis of *JAG1 and THBS2* genes in a family. Semifilled symbol indicates heterozygous status for *JAG1* mutation (gray color) (p.Pro943Leu) or *THBS2* mutation (black color) (p.Pro1099Leu). DNA sequence analysis was performed in each individual reported here, after obtaining informed consent in accordance with protocols for human studies approved by our medical center.

We report on a boy who had neonatal cholestasis with elevated serum GGT activity (308 IU/L). At age of 2 months, cholangiography excluded biliary atresia, liver histology showed severe ductopenia, and cardiac ultrasonography a patent foramen ovale. Other target organs were not affected. Genetic analysis identified in the boy a maternal heterozygous *JAG1* mutation (NM_000214; c.2828C>T; p.Pro943Leu; ACMG classification: class 3; gnomAD 0.00438%) and a paternal heterozygous *THBS2* mutation (NM_003247; c.3296C>T; p.Pro1099Leu; class 2; gnomAD 0.009%) (Fig. 1). His parents were healthy but the mother who transmitted the *JAG1* mutation refused any clinical investigation. This observation in a child with incomplete ALGS affecting only the liver and heart, together with data from the literature (2), further suggests that *THBS2* could be a modifier gene in some ALGS patients with *JAG1* mutations and could play a role in the variable expressivity of this syndrome. The combination of both mutants could explain the incomplete ALGS phenotype in the propositus, by disrupting the JAG1–NOTCH2 signaling. However, a functional evaluation of the interaction of a THBS2 mutant on the Notch signaling pathway as well as the search for *THBS2* mutations in patients with ALGS are needed to conclude that *THBS2* can be a modifier gene in ALGS.

## ACKNOWLEDGMENTS

The family is aware of the intent to publish and agreed to it. We thank the Association Maladie Foie Enfants (AMFE), Malakoff, France, Association “Pour Louis 1000 Foie Merci” (Fournet-Luisans, France), and Fondation Rumsey-Cartier (Genève, Switzerland) for their support.
